# Supplementary figures and images for: Mutations in the Staphylococcus aureus Global Regulator CodY confer tolerance to an interspecies redox-active antimicrobial
Source: PLoS Genet. 2025 Mar 7;21(3):e1011610. doi: 10.1371/journal.pgen.1011610 (PMC11918324; doi:10.1371/journal.pgen.1011610)

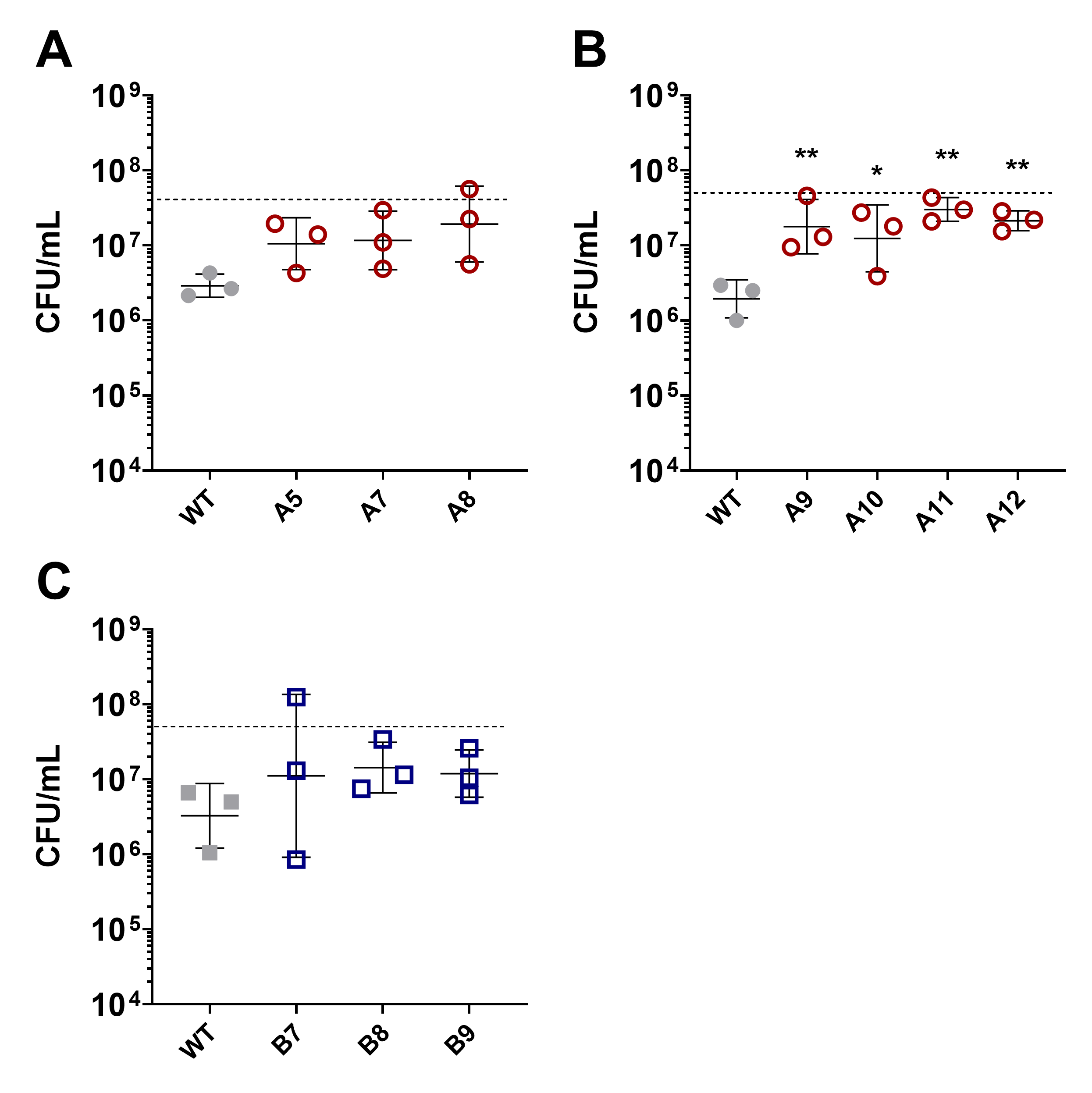

Supplement: S1 Fig — (TIF) [file pgen.1011610.s001.tif]

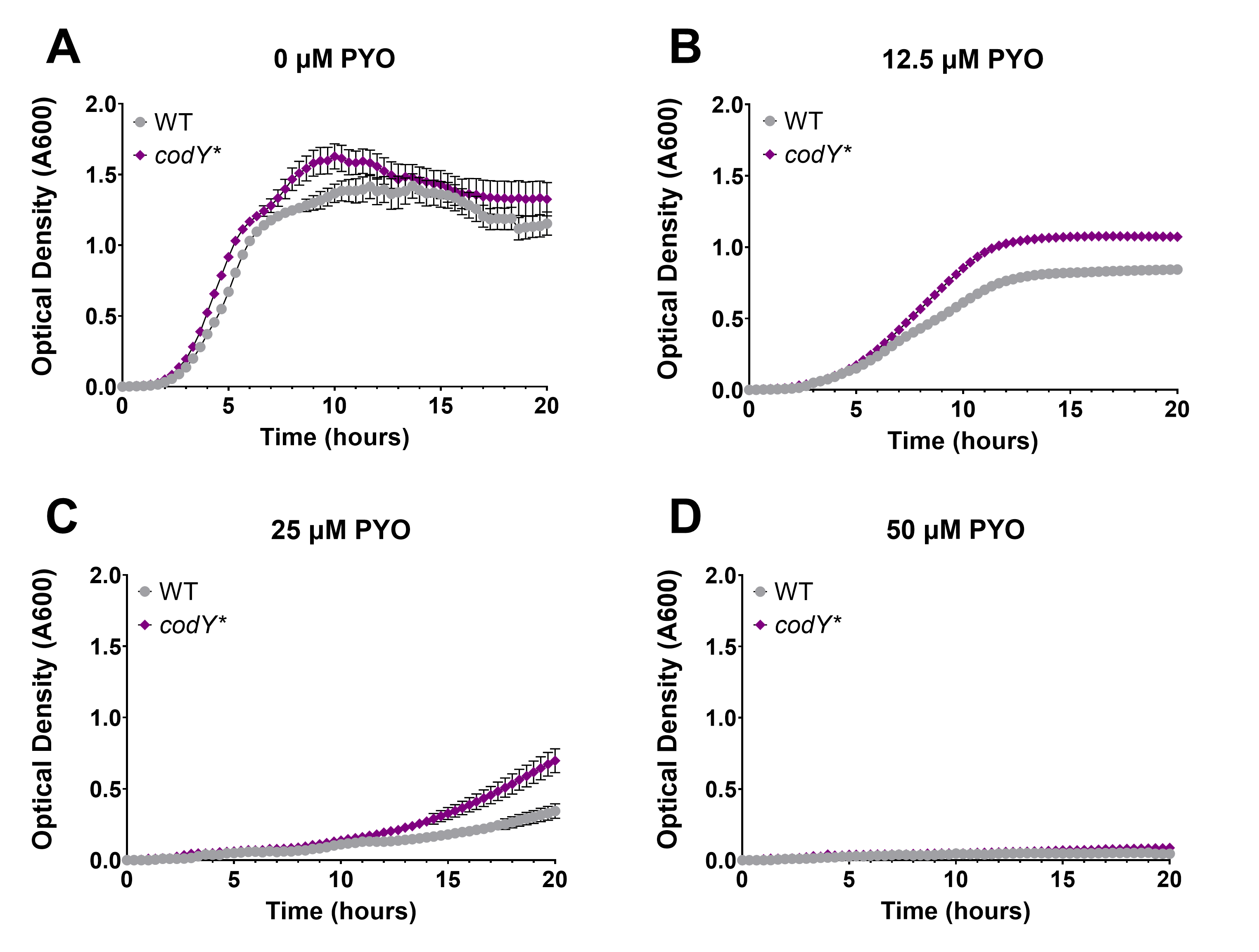

Supplement: S2 Fig — (TIF) [file pgen.1011610.s002.tif]

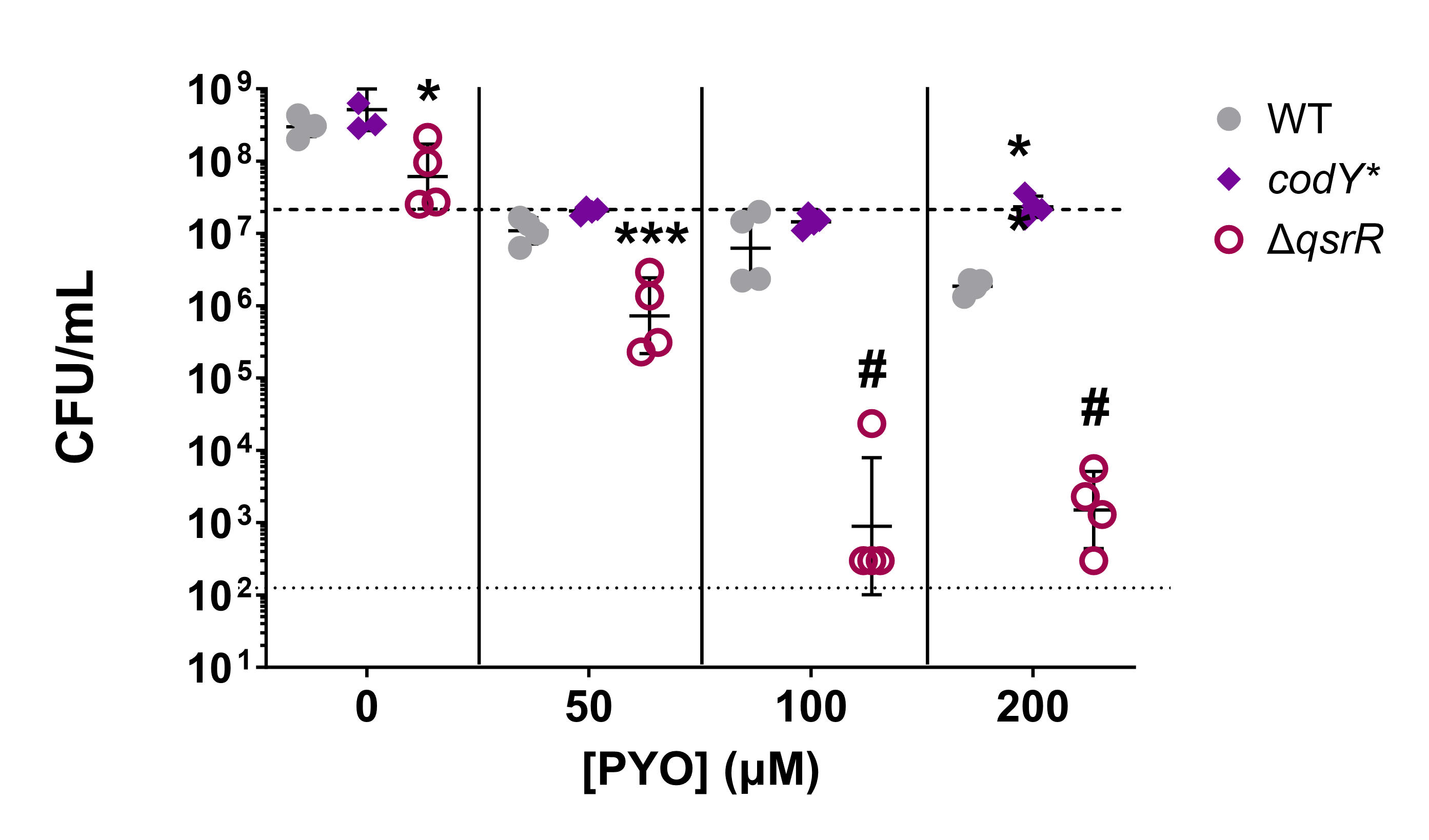

Supplement: S3 Fig — (TIF) [file pgen.1011610.s003.tif]

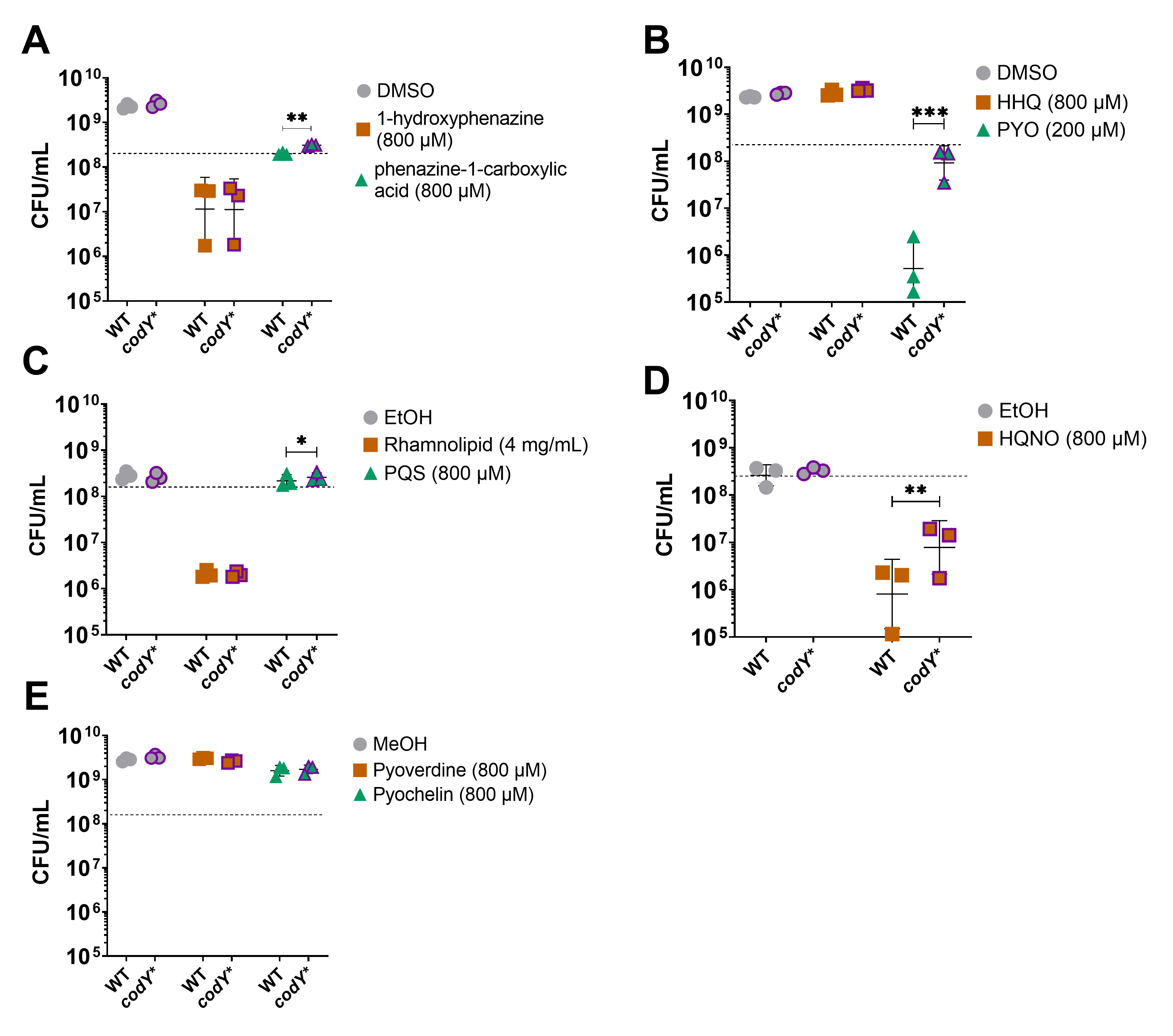

Supplement: S4 Fig — (TIF) [file pgen.1011610.s004.tif]

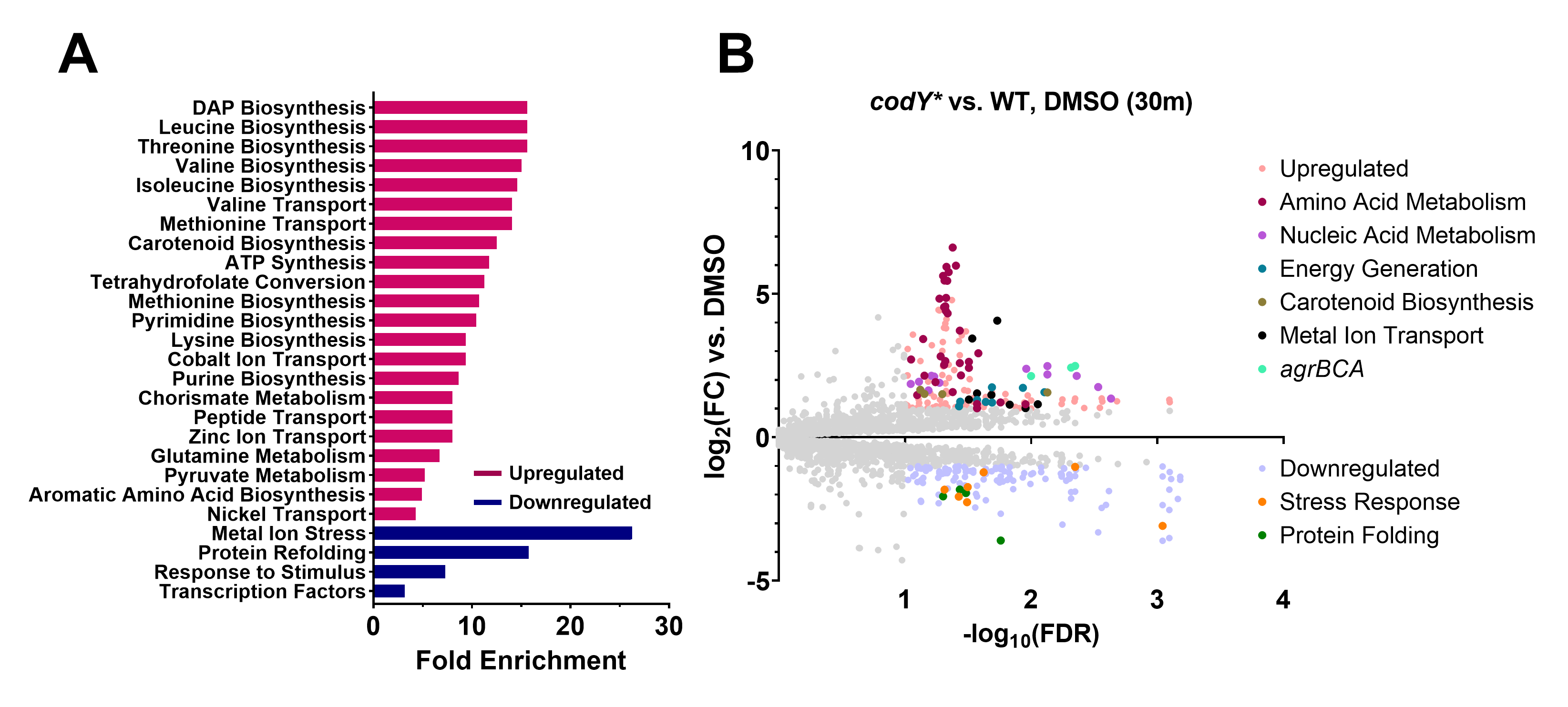

Supplement: S5 Fig — (TIF) [file pgen.1011610.s005.tif]

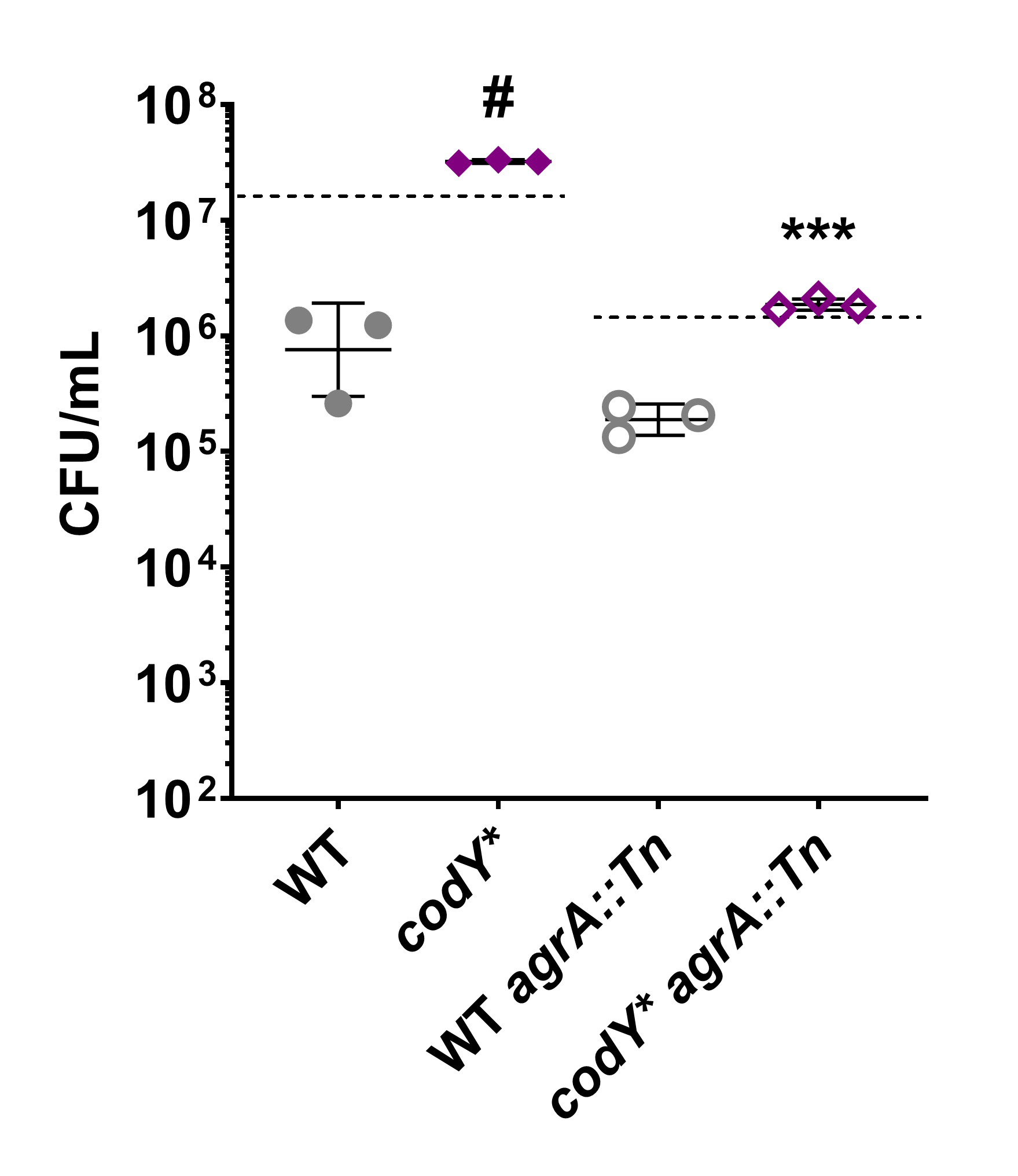

Supplement: S6 Fig — (TIF) [file pgen.1011610.s006.tif]

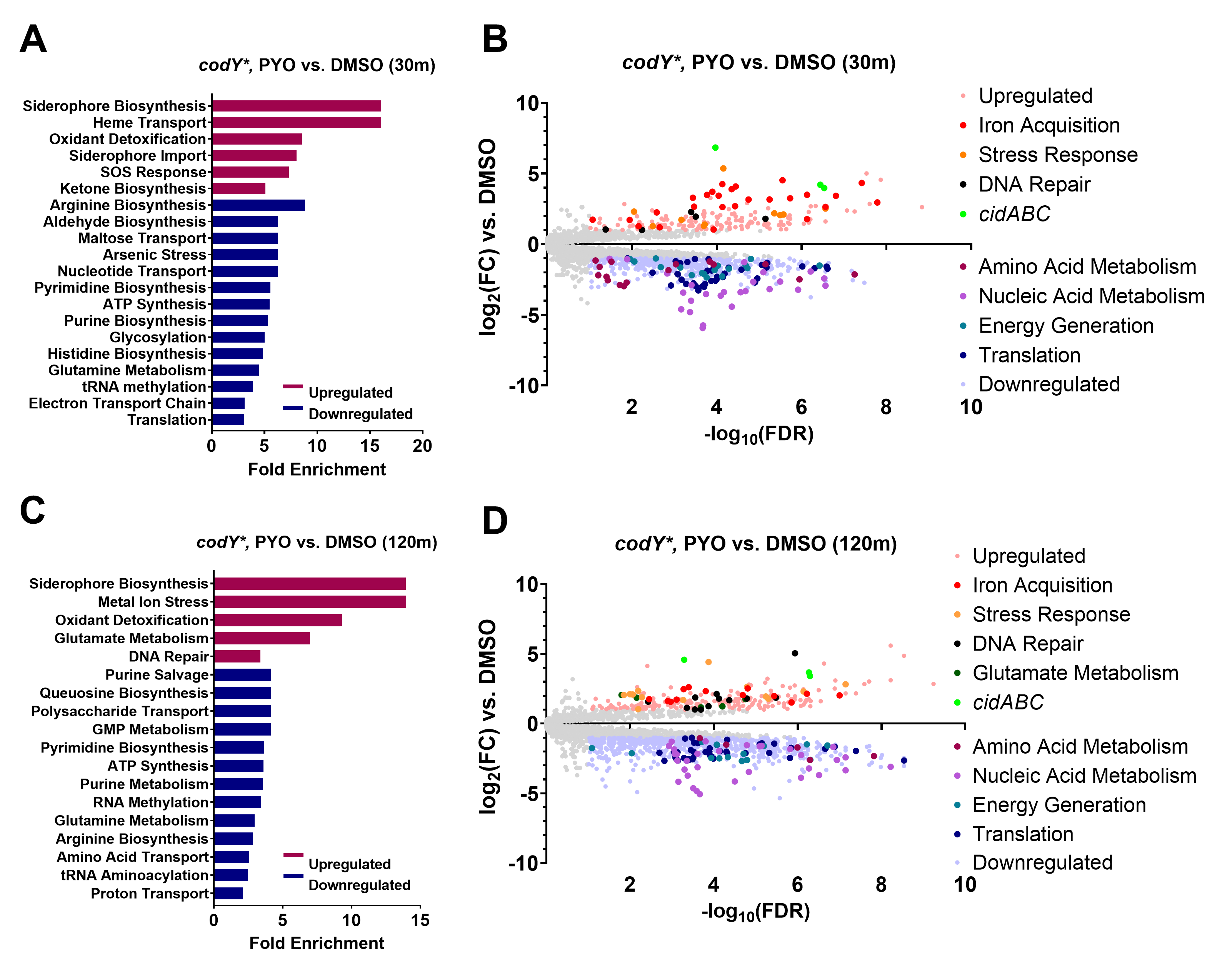

Supplement: S7 Fig — (TIF) [file pgen.1011610.s007.tif]

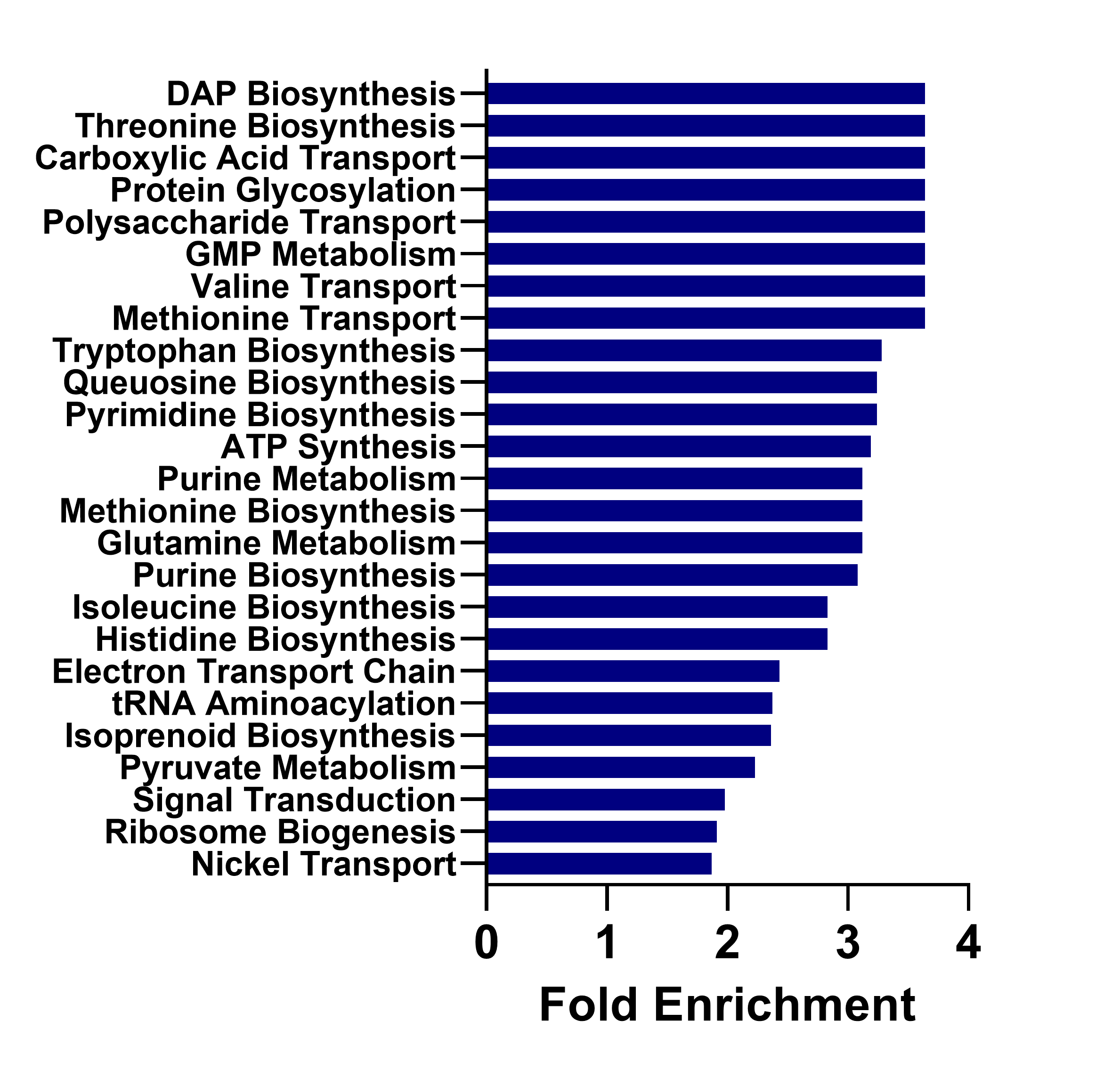

Supplement: S8 Fig — (TIF) [file pgen.1011610.s008.tif]

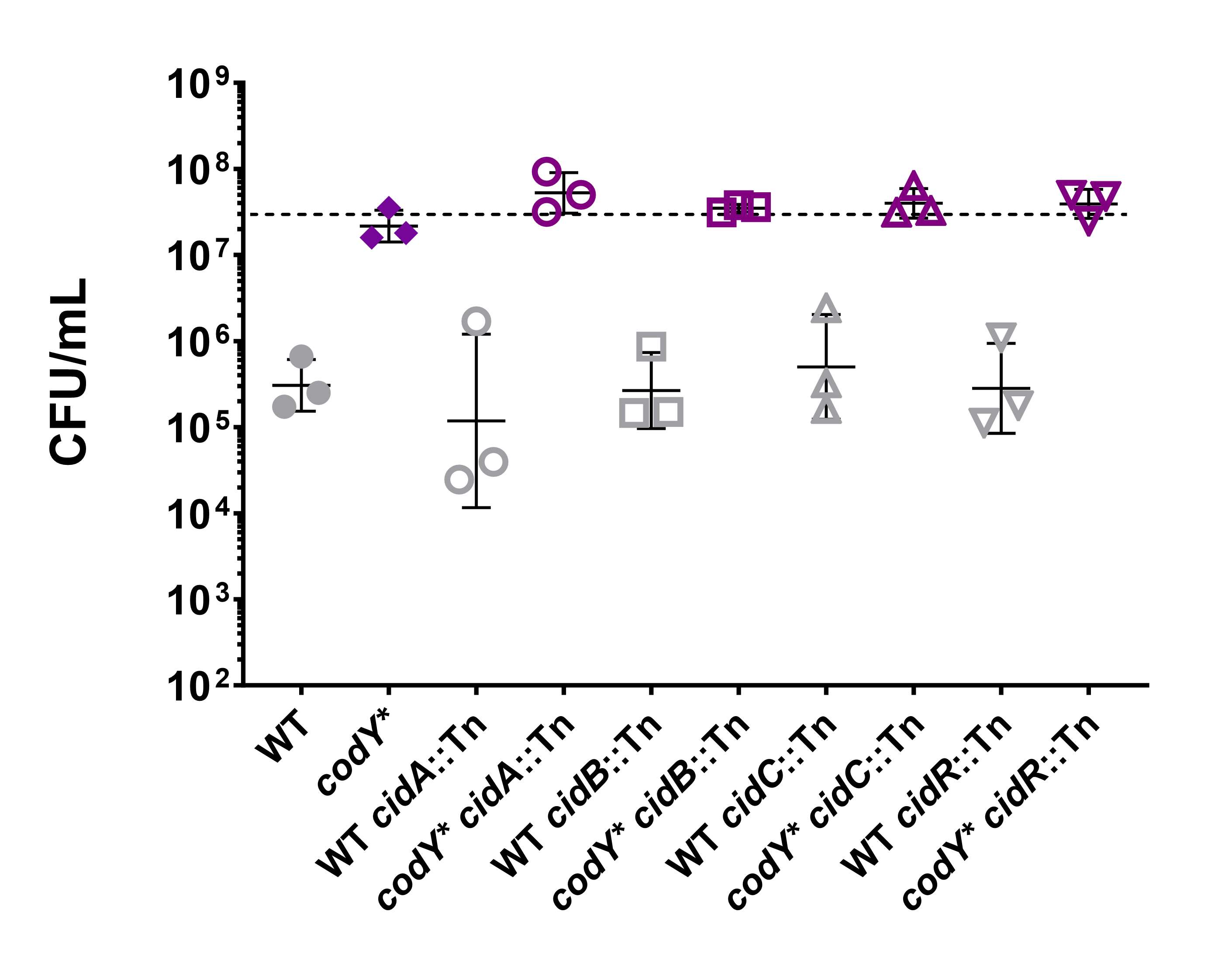

Supplement: S9 Fig — (TIF) [file pgen.1011610.s009.tif]

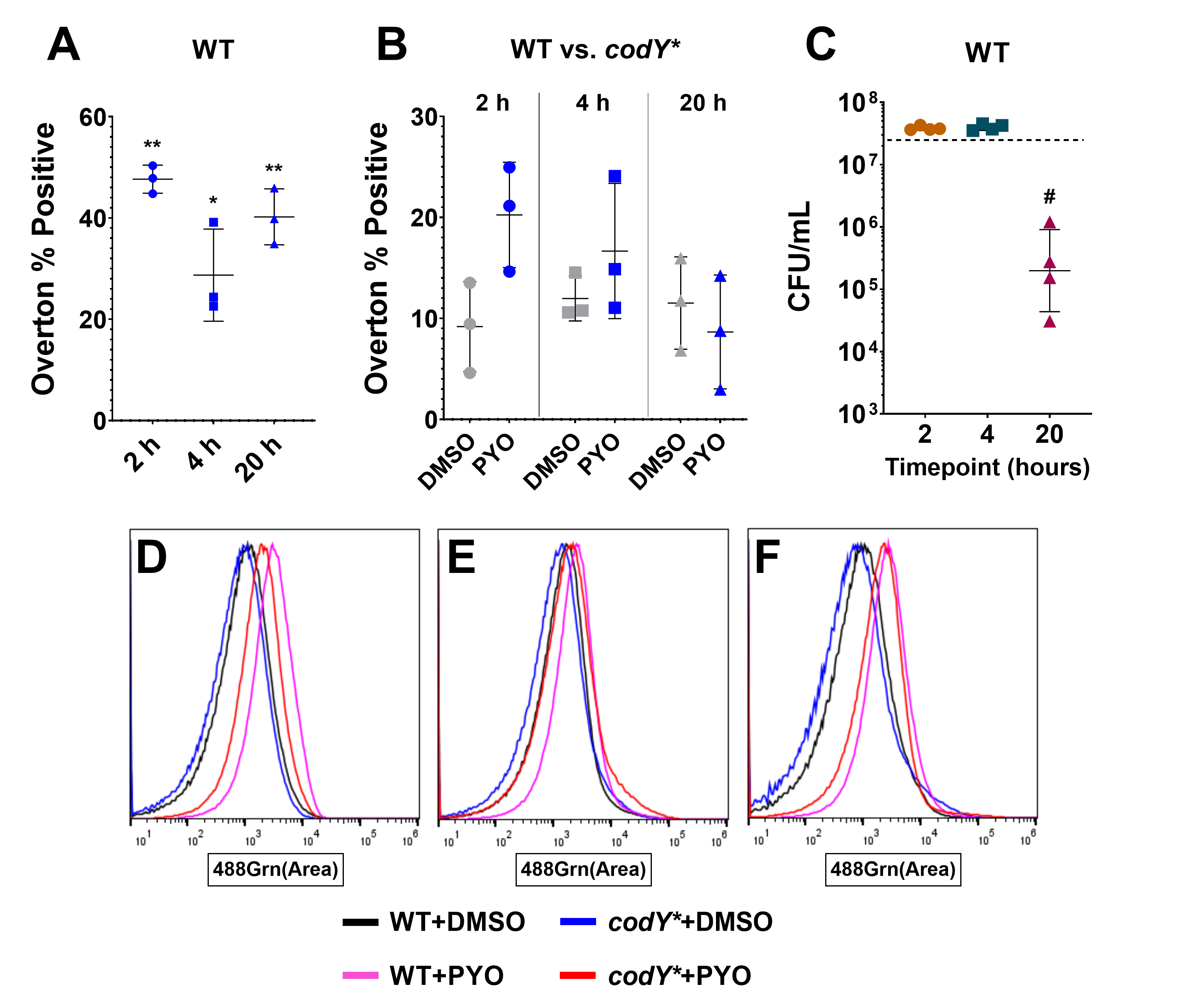

Supplement: S10 Fig — (TIF) [file pgen.1011610.s010.tif]

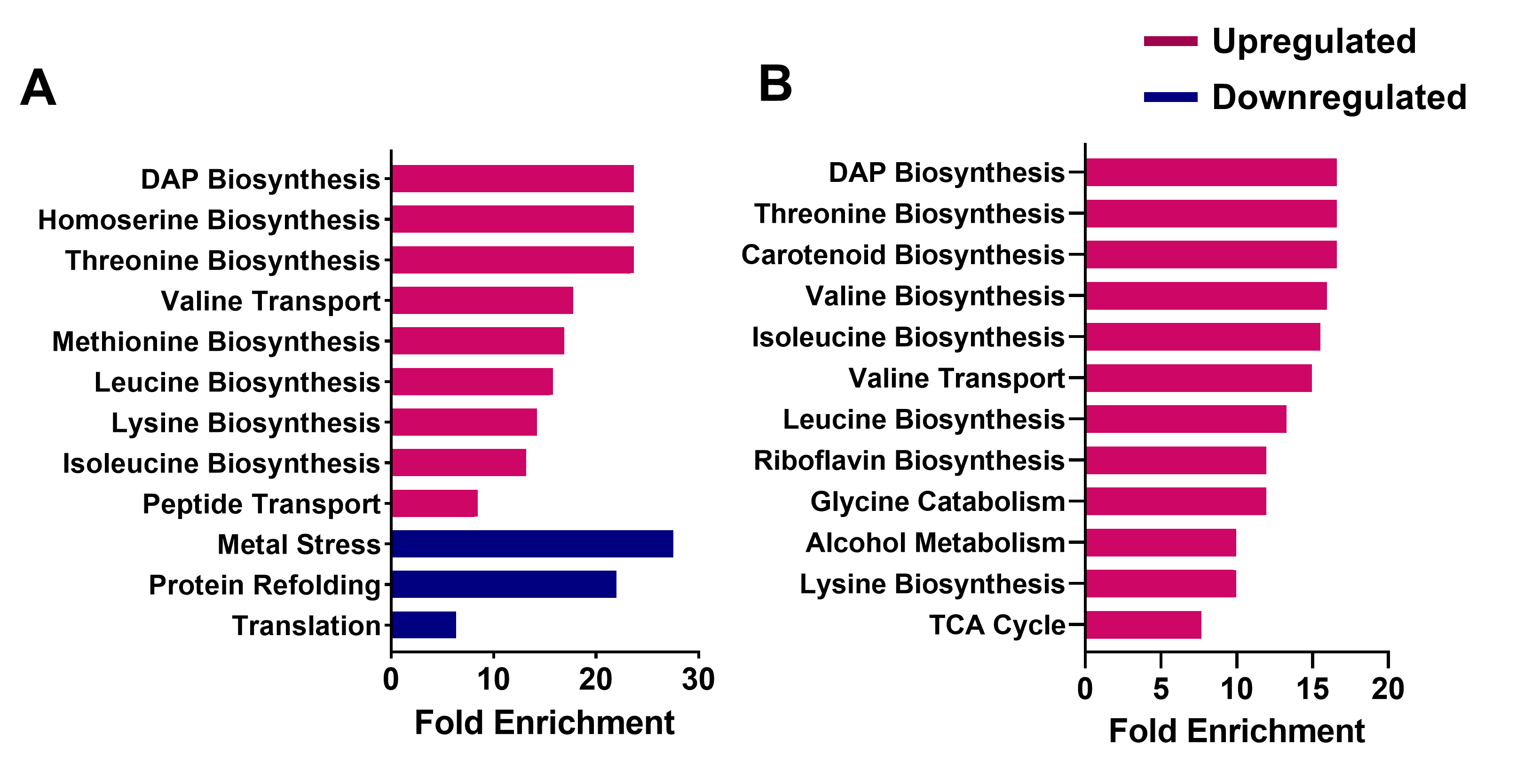

Supplement: S11 Fig — (TIF) [file pgen.1011610.s011.tif]

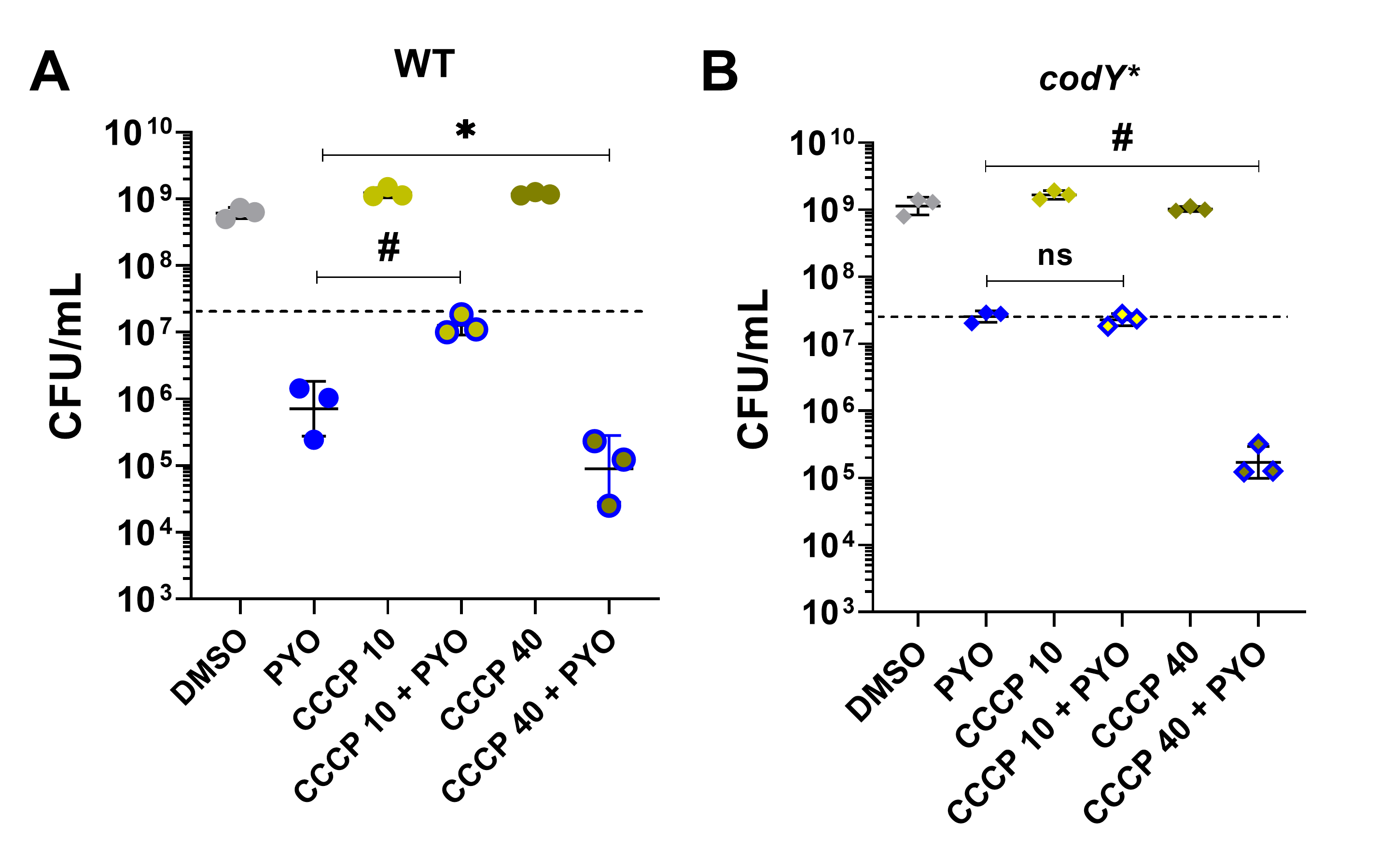

Supplement: S12 Fig — (TIF) [file pgen.1011610.s012.tif]

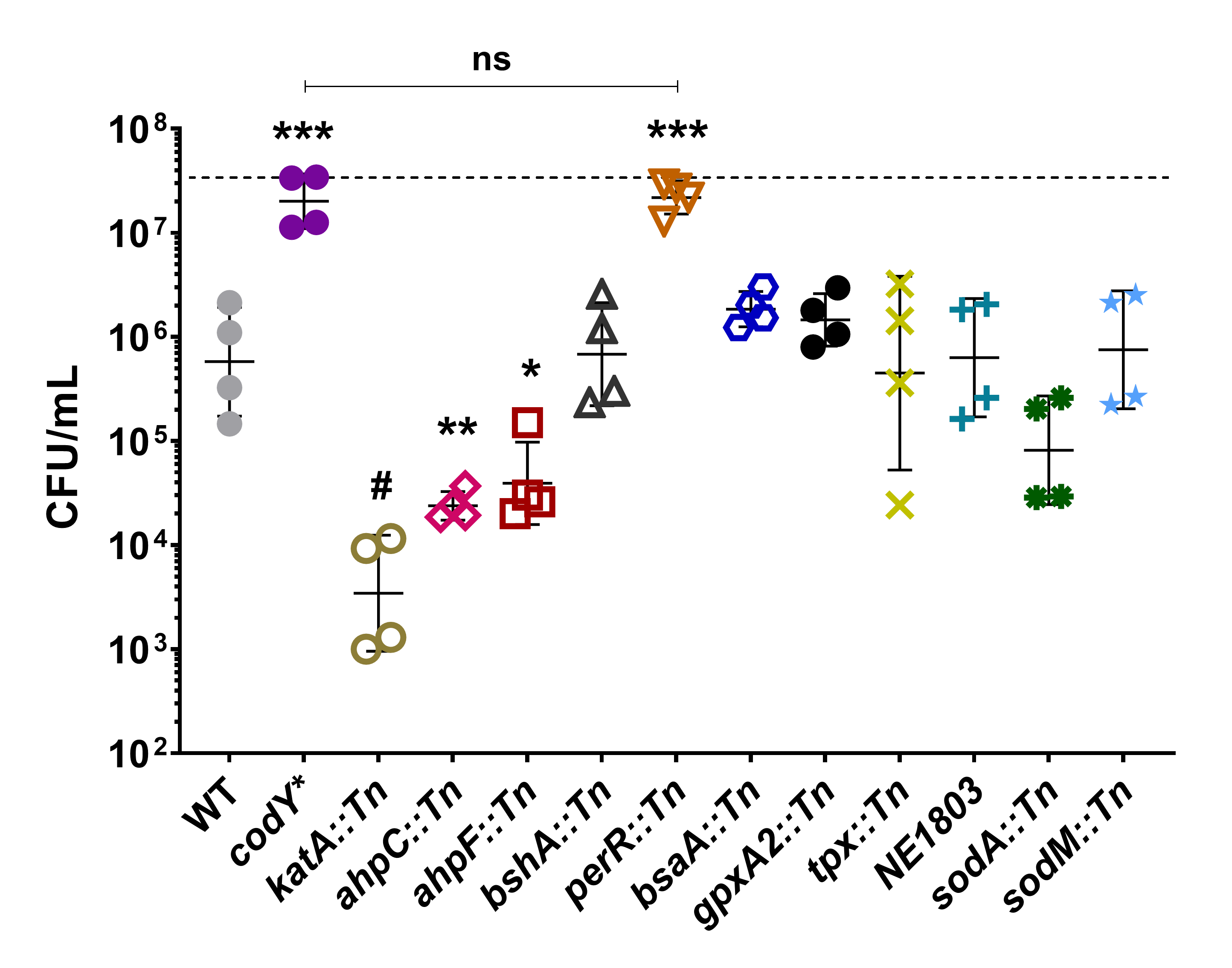

Supplement: S13 Fig — (TIF) [file pgen.1011610.s013.tif]

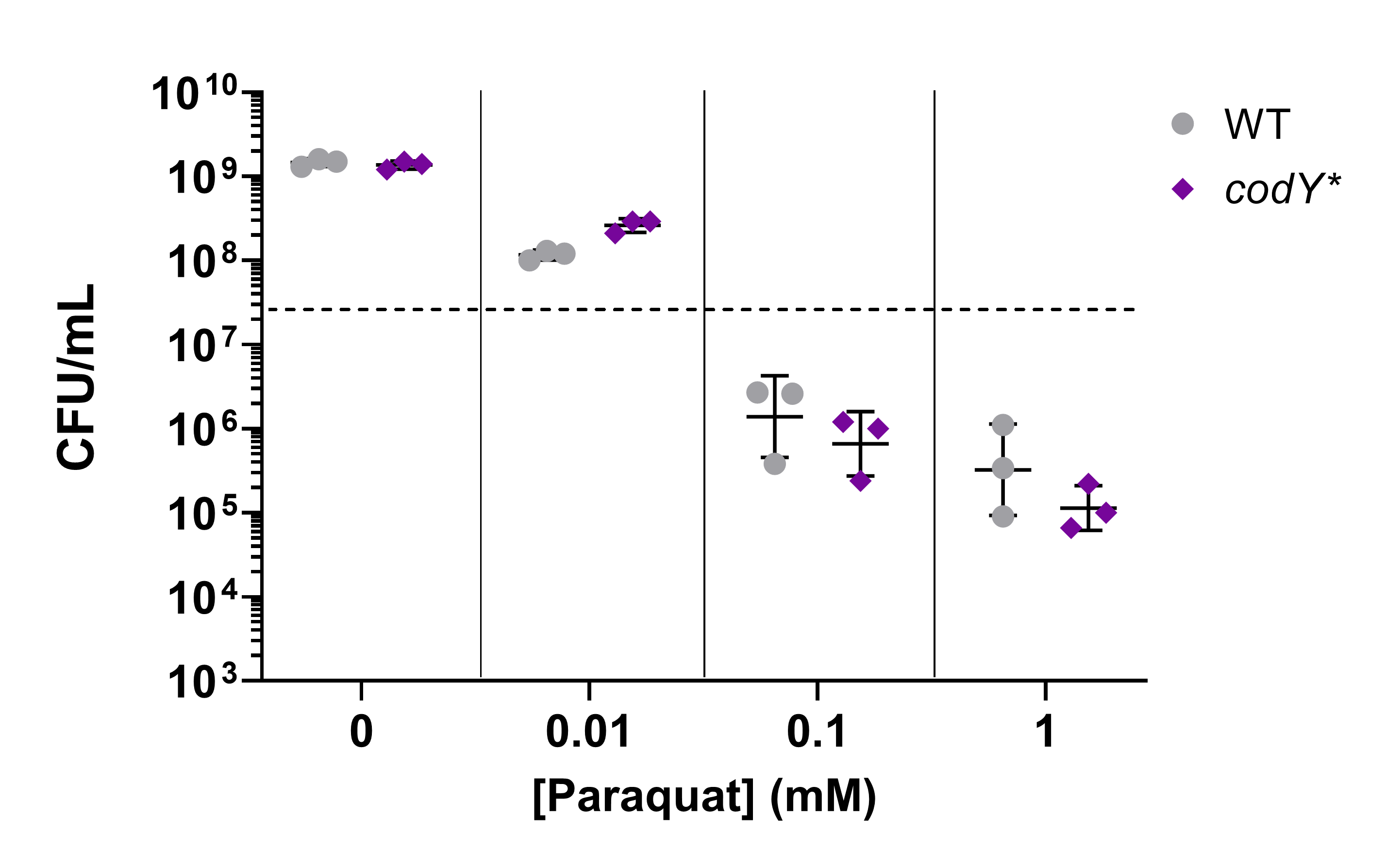

Supplement: S14 Fig — (TIF) [file pgen.1011610.s014.tif]

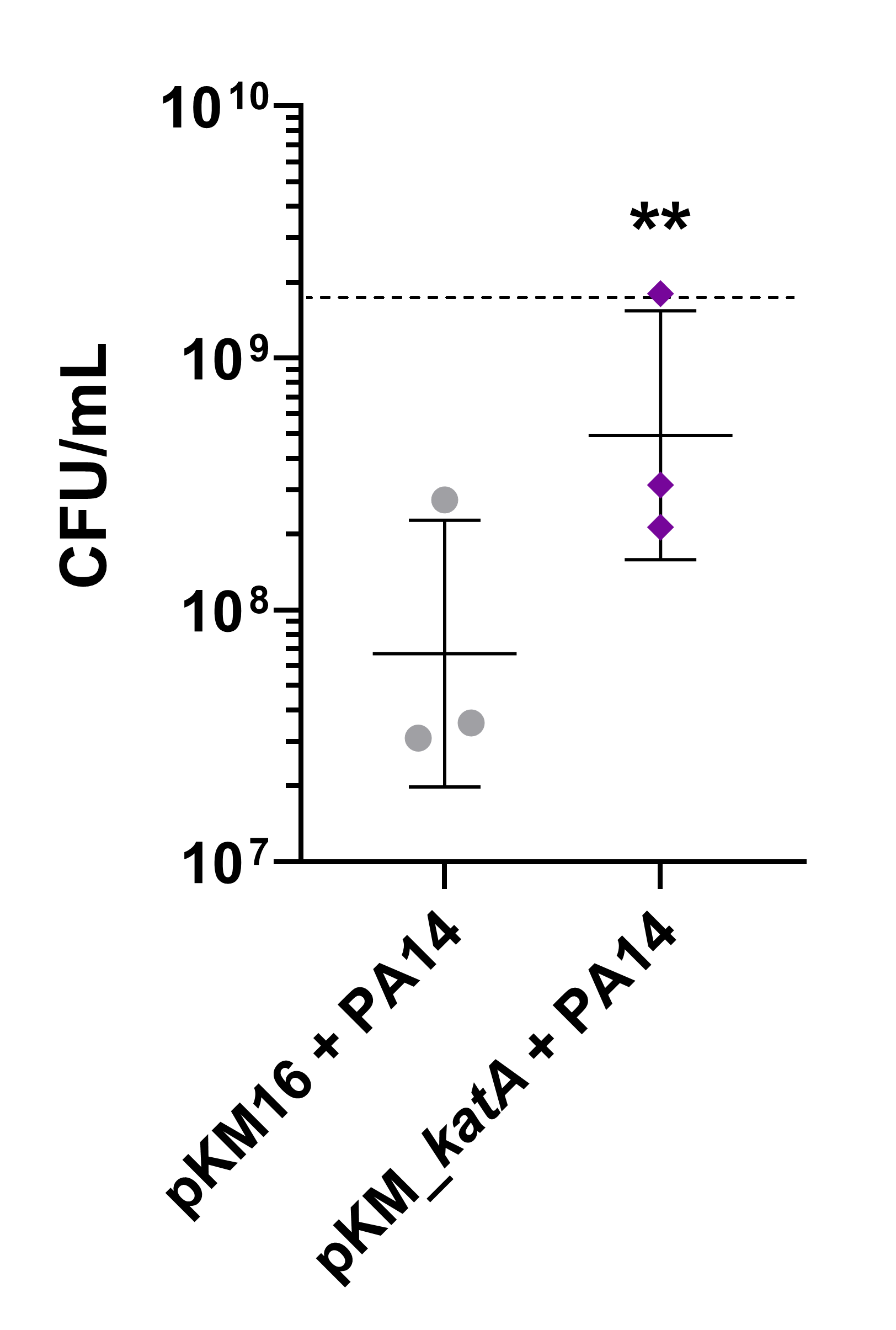

Supplement: S15 Fig — (TIF) [file pgen.1011610.s015.tif]
