# Supplementary material for: Mutations in the Staphylococcus aureus Global Regulator CodY confer tolerance to an interspecies redox-active antimicrobial
Source: PLoS Genet. 2025 Mar 7;21(3):e1011610. doi: 10.1371/journal.pgen.1011610 (PMC11918324; doi:10.1371/journal.pgen.1011610)
Supplement: S1 Table — (PDF) [file pgen.1011610.s016.pdf]

S1 Table. CodY-associated mutations in PYO-evolved isolates.

Designation	Isolate	CodY Mutation
		**Population A**
200.5-A-1	A1	Intergenic (+15/-9)<br>AG → GT
200.5-A-2	A2	Intergenic (+15/-9)<br>AG → GT
200.5-A-3	A3	R222C
200.5-A-4	A4	S178L
200.5-A-5	A5	T125I
200.5-A-7	A7	R61K
200.5-A-8	A8	Intergenic (+15/-9)<br>AG → GT
200.5-A-9	A9	R222C
200.5-A-10	A10	Intergenic (+15/-9)<br>AG → GT
200.5-A-11	A11	Intergenic (+15/-9)<br>AG → GT
200.5-A-12	A12	Y75C
		**Population B**
200.7-B-1	B1	G118D
200.7-B-3	B3	Intergenic (+16/-9)<br>G → T
200.7-B-4	B4	G118D
200.7-B-5	B5	G118D
200.7-B-7	B7	ATG → ATA
200.7-B-8	B8	G118D
200.7-B-9	B9	G118D
