# Supplementary material for: Mutations in the Staphylococcus aureus Global Regulator CodY confer tolerance to an interspecies redox-active antimicrobial
Source: PLoS Genet. 2025 Mar 7;21(3):e1011610. doi: 10.1371/journal.pgen.1011610 (PMC11918324; doi:10.1371/journal.pgen.1011610)
Supplement: S2 Table — (PDF) [file pgen.1011610.s017.pdf]

S2 Table. Strains and plasmids used in this study.

Name	Description	Source
**Strains**		
Staphylococcus aureus		
JE2	Plasmid-cured derivative of the USA300 strain, LAC	[1,2]
RN4220	Restriction-deficient mutant of NCTC8325-4	[3]
SB523	JE2 *codY*^{R222C}	This study
NE1555	*codY*::Tn	[2]
SB524	JE2 Δ*qsrR*	This study
NE1532	*agrA*::Tn	[2]
SB525	*agrA*::Tn *codY*^{R222C}	This study
NE1366	*katA*::Tn	[2]
SB526	*katA*::Tn *codY*^{R222C}	This study
NE911	*ahpC*::Tn	[2]
SB527	*ahpC*::Tn *codY*^{R222C}	This study
NE1571	*ahpF*::Tn	[2]
NE1728	*bshA*::Tn	[2]
NE665	*perR*::Tn	[2]
NE390	*gltB*::Tn	[2]
SB529	*gltB*::Tn *codY*^{R222C}	This study
NE445	*umuC*::Tn	[2]
SB530	*umuC*::Tn *codY*^{R222C}	This study
NE563	*gpxA2*::Tn	[2]
SB531	*gpxA2*::Tn *codY*^{R222C}	This study
NE1730	*bsaA*::Tn	[2]
SB532	*bsaA*::Tn *codY*^{R222C}	This study
NE1803	RS04260::Tn	[2]
NE1332	*tpx*::Tn	[2]
NE1932	*sodA*::Tn	[2]
NE1224	*sodM*::Tn	[2]
NE122	*adhC*::Tn	[2]
SB533	*adhC*::Tn *codY*^{R222C}	This study
NE1538	*crtO*::Tn	[2]
SB534	*crtO*::Tn *codY*^{R222C}	This study
NE1692	*cidA*::Tn	[2]
SB536	*cidA*::Tn *codY*^{R222C}	This study
NE935	*cidB*::Tn	[2]
SB537	*cidB*::Tn *codY*^{R222C}	This study
NE564	*cidC*::Tn	[2]
SB538	*cidC*::Tn *codY*^{R222C}	This study
NE1466	*cidR*::Tn	[2]
SB539	*cidR*::Tn *codY*^{R222C}	This study
Pseudomonas aeruginosa		
PA14	University of California Plant Pathology (UCBPP)-PA14	[4]
AK681	PA14 Δ*phz1* Δ*phz2*	[5]

AK732	PA14 Δ*phzM*	This study
Escherichia coli		
DC10B	Cloning *E. coli* DC10B, *dam*/*dcm*-deficient	[6]
SB192	Cloning *E. coli* IM08B with USA300-pattern restriction modification	[7]
S17-1 λ-pir	Conjugative strain	[8]
**Plasmids**		
pIMAY*	Allelic exchange vector; Cm^R	[9]
pIMAY*-*codY*^{R222C}	Allelic exchange vector for construction of the *codY** allele	This study
SB209	pKM16; fluorescent reporter; Cm^R	[10]
SB540	pKM16 backbone expressing *katA* from its native promoter	This study
SB541	pKM16 backbone expressing *ahpCF* from its native promoter	This study
SB542	pKM16 backbone expressing *umuC* from its native promoter	This study
SB543	pKM16 backbone expressing *pxpBCA* from its native promoter	This study
SB544	pKM16 backbone expressing *adhC* from its native promoter	This study
PCR8/GW/TOPO	Gateway entry vector; $Spec^R$	Invitrogen
PCR8/GW/TOPO-Δ*phzM*	Gateway Entry vector with the *phzM* deletion construct	This study
pEX18ApGW	Gateway-compatible gene replacement vector; Amp^R Cm^R	[11]
pEX18ApGW-Δ*phzM*	Gene replacement vector with the *phzM* deletion construct	This study
pFLP2	FLP recombinase expressing plasmid; Amp^R $Carb^R$	[12]

REFERENCES

1. Miller LG, Rieg G, Bayer AS, Spellberg B. Necrotizing Fasciitis Caused by Community-Associated Methicillin-Resistant *Staphylococcus aureus* in Los Angeles. N Engl J Med. 2005; 9.

2. Fey PD, Endres JL, Yajjala V, Widhelm TJ, Boissy RJ, Bose JL, et al. A Genetic Resource for Rapid and Comprehensive Phenotype Screening of Nonessential *Staphylococcus aureus* Genes. mBio. 2013;4: e00537-12. doi:10.1128/mBio.00537-12

3. Iordanescu S, Surdeanu M. Two Restriction and Modification Systems in *Staphylococcus aureus* NCTC8325. J Gen Microbiol. 1976;96: 277–281. doi:10.1099/00221287-96-2-277

4. Rahme LG, Stevens EJ, Wolfort SF, Shao J, Tompkins RG, Ausubel FM. Common Virulence Factors for Bacterial Pathogenicity in Plants and Animals. Science. 1995;268: 1899–1902. doi:10.1126/science.7604262

5. Khare A, Tavazoie S. Multifactorial Competition and Resistance in a Two-Species Bacterial System. PLoS Genet. 2015;11: e1005715. doi:10.1371/journal.pgen.1005715

6. Monk IR, Shah IM, Xu M, Tan M-W, Foster TJ. Transforming the Untransformable: Application of Direct Transformation To Manipulate Genetically *Staphylococcus aureus* and *Staphylococcus epidermidis*. mBio. 2012;3: e00277-11. doi:10.1128/mbio.00277-11

7. Monk IR, Tree JJ, Howden BP, Stinear TP, Foster TJ. Complete Bypass of Restriction Systems for Major *Staphylococcus aureus* Lineages. mBio. 2015;6: e00308-15. doi:10.1128/mBio.00308-15

8. De Lorenzo V, Timmis KN. Analysis and construction of stable phenotypes in gram-negative bacteria with Tn5- and Tn10-derived minitransposons. Methods in Enzymology. Elsevier; 1994. pp. 386–405. doi:10.1016/0076-6879(94)35157-0

9. Schuster CF, Howard SA, Gründling A. Use of the counter selectable marker PheS* for genome engineering in *Staphylococcus aureus*. Microbiol. 2019;165: 572–584. doi:10.1099/mic.0.000791

10. Mlynek KD, Bulock LL, Stone CJ, Curran LJ, Sadykov MR, Bayles KW, et al. Genetic and Biochemical Analysis of CodY-Mediated Cell Aggregation in *Staphylococcus aureus* Reveals an Interaction between Extracellular DNA and Polysaccharide in the Extracellular Matrix. J Bacteriol. 2020;202. doi:10.1128/JB.00593-19

11. Choi K-H, Schweizer HP. An improved method for rapid generation of unmarked *Pseudomonas aeruginosa* deletion mutants. BMC Microbiol. 2005;5: 30. doi:10.1186/1471-2180-5-30

12. Hoang TT, Karkhoff-Schweizer RR, Kutchma AJ, Schweizer HP. A broad-host-range Flp-FRT recombination system for site-specific excision of chromosomally-located DNA sequences: application for isolation of unmarked *Pseudomonas aeruginosa* mutants. Gene. 1998;212: 77–86. doi:10.1016/S0378-1119(98)00130-9
