# Supplementary material for: Mutations in the Staphylococcus aureus Global Regulator CodY confer tolerance to an interspecies redox-active antimicrobial
Source: PLoS Genet. 2025 Mar 7;21(3):e1011610. doi: 10.1371/journal.pgen.1011610 (PMC11918324; doi:10.1371/journal.pgen.1011610)
Supplement: S3 Table — (PDF) [file pgen.1011610.s018.pdf]

S3 Table. Oligonucleotides used in this study.

Primer Name	Description	Primer Sequence*
Sa035	Forward amplification of *codY* adding an EcoRI site with homologous sequence to pIMAY* for Gibson assembly	GGTATCGATAAGCTTGATATC**GAATTC**CGACAAAGTTGCGACGAATA
Sa036	Reverse amplification of *codY* adding a NotI site with homologous sequence to pIMAY* for Gibson assembly	TGGAGCTCCACCGCGGTG**GCGGCCGC**ATCACGAATTCCGCCTAAGA
Sa037	Forward pIMAY*-*codY* integration primers	AAGAAAGTTGAGCGAGAA
Sa038	Reverse pIMAY*-*codY* integration primers	AATTGCATTACGCTCTTT
JE2/qsrR/Up.F1	Upstream fragment for *qsrR* allelic exchange	TCACTAAAGGGAACAAAAGCTCTTTAACAACTAAATCATCTCCG
JE2/qsrR/Up.R1	Upstream fragment for *qsrR* allelic exchange	AGATGATGGAAGTATGTCCGCGTACTGCTAAATAATTACATGACG
IMAY*/qsrR/Up.F2	Vector linearization for *qsrR* allelic exchange	GCCTGCTTCAATTCTATCAGCGTCACAGGTATTTATTCGG
IMAY*/qsrR/Up.R2	Vector linearization for *qsrR* allelic exchange	GATGATTTAGTTGTTAAAGAGCTTTTGTTCCCTTTAGTGA
JE2/qsrR/Down.F3	Downstream fragment for *qsrR* allelic exchange	TGTAATTATTTAGCAGTACGCGGACATACTTCCATCATCTTC
JE2/qsrR/Down.R3	Downstream fragment for *qsrR* allelic exchange	CCGAATAAATACCTGTGACGCTGATAGAATTGAAGCAGGC
NTML/Buster2.R	Transposon primer (reverse)	GCCAACCTGTTACTAGACCG
NTML/Upstream2.F	Transposon primer (forward)	AAAGCATTGAACACCATAACCG
JE2/agrA/NTML.R1	Screening of *agrA* transposon insertion	TACACTGAATTACTGCCACG
JE2/cidA/NTML.R1	Screening of *cidA* transposon insertion	GAAAATGAAGTGAAATTTAGAGAGC
JE2/cidB/NTML.F1	Screening of *cidB* transposon insertion	TCATCCCAATTGAACTAAATGC
JE2/cidC/NTML.F1	Screening of *cidC* transposon insertion	GTTTAAGAATGGTCTTTCAGCA
JE2/cidR/NTML.R1	Screening of *cidR* transposon insertion	GAAATAGTTAGGATGATGTTAGTGG
JE2/katA/NTML.F1	Screening of *katA* transposon insertion	GCAGCTTGTTCAACATCC
JE2/ahpC/NTML.R1	Screening of *ahpC* transposon insertion	CATTACCTTCATCCATCTCG
JE2/ahpF/NTML.F1	Screening of *ahpF* transposon insertion	GCACGTATACCTGTCATTGC
JE2/perR/NTML.F1	Screening of *perR* transposon insertion	ATCATTGCGACAAGCAGG
JE2/gltB/NTML.F1	Screening of *gltB* transposon insertion	CTTGAAATGTTGCGACGC

JE2/gpxA2/NTML.F1	Screening of *gpxA2* transposon insertion	TCATTAGAACCTGGTTGTCG
JE2/bshA/NTML.F1	Screening of *bshA* transposon insertion	GATCATTCACTCCAAGGTGC
JE2/tpx/NTML.F1	Screening of *tpx* transposon insertion	ATAACATTCAAAGGTGGACC
JE2/umuC/NTML.F1	Screening of *umuC* transposon insertion	GTTGTTGCAGATACTAAGCG
JE2/RS04260/NTML.R1	Screening of *RS04260* transposon insertion	TTTATGGATTGACCCAATCG
JE2/crtO/NTML.R1	Screening of *crtO* transposon insertion	TCTCTAACATGAGAGTATTGGC
JE2/adhC/NTML.R1	Screening of *adhC* transposon insertion	ATACCTCCTAAACCAACAACCGC
pKM16/Dn/Linear.F1	Linearization of pKM16 excluding *sarA_dsRed*	CGGTTATCCACAGAATCAGG
pKM16/Up/Linear.R1	Linearization of pKM16 excluding *sarA_dsRed*	AAAGATCCTAACGAAAAGCG
JE2/umuC/Up.F1	*umuC* upstream primer for pKM_umuC	CGCTTTTCGTTAGGATCTTTCGATTGGC AACATCCAAACC
JE2/umuC/Dn.R1	*umuC* downstream primer for pKM_umuC	CCTGATTCTGTGGATAACCGAAACCCTA CTGACCGAGAAC
JE2/katA/Up.F1	*katA* upstream primer for pKM_umuC	CGCTTTTCGTTAGGATCTTTTAAAATGTT GCCAACTCTCC
JE2/katA/Dn.R1	*katA* downstream primer for pKM_umuC	CCTGATTCTGTGGATAACCGCATAAACT GCTCAACTACGC
JE2/ahpCF/Up.F1	*ahpCF* upstream primer for pKM_umuC	CGCTTTTCGTTAGGATCTTTATCTTCTCA TCGTCGATACC
JE2/ahpCF/Dn.R1	*ahpCF* downstream primer for pKM_umuC	CCTGATTCTGTGGATAACCGAAGCATTAT CGCACATCTCG
JE2/pxpA/Up.F1	*pxpA* upstream primer for pKM_umuC	CCTGATTCTGTGGATAACCGTTCTCCCC ATTTTTTTAGCC
JE2/pxpA/Dn.R1	*pxpA* downstream primer for pKM_umuC	GAAAAAATCGATGCGAGTTGATTTGAATT G
JE2/pxpBC/Up.F1	*pxpBC* upstream primer for pKM_umuC	CAACTCGCATCGATTTTTTCAATATTGAT TTTACAAATCC
JE2/pxpBC/Dn.R1	*pxpBC* downstream primer for pKM_umuC	CGCTTTTCGTTAGGATCTTTGAATCACCA ATGGCTAAAGC
JE2/adhC/Up.F1	*adhC* upstream primer for pKM_umuC	CGCTTTTCGTTAGGATCTTTAGTTTCATA ATCCCACTCCC
JE2/adhC/Dn.R1	*adhC* downstream primer for pKM_umuC	CCTGATTCTGTGGATAACCGGAGCATCC TTCACTTTTGCG
Gm-F	Forward primer for FRT-flanked gentamycin-resistance cassette	CGAATTAGCTTCAAAAGCGCTCTGA
Gm-R	Reverse primer for FRT-flanked gentamycin-resistance cassette	CGAATTGGGGATCTTGAAGTTCCT
phzM_up_F	Forward primer for upstream fragment of *phzM*	GCACTAGTCTGGCGCATTGTTCATAC

phzM_up_R	Reverse primer for upstream fragment of *phzM*	TCAGAGCGCTTTTGAAGCTAATTCGTATCAAATTACGCGCAGCAG
phzM_down_F	Forward primer for downstream fragment of *phzM*	AGGAACTTCAAGATCCCCAATTCGGTCGACCTGCCGATGGAAAC
phzM_down_R	Reverse primer for downstream fragment of *phzM*	GCACTAGTCGCCCCGACGTAGTTAGC
phzM_test_F	Screening of *phzM* deletion	GAGGGCTCTCCAGGTATGC
phzM_test_R	Screening of *phzM* deletion	GCCATTGGCGAAGAACCTG

***Bold indicates the restriction cut site.**

**Underlined sequences indicate regions of homology for Gibson assembly or overlap extension PCR.
