# Supplementary material for: Mutations in the Staphylococcus aureus Global Regulator CodY confer tolerance to an interspecies redox-active antimicrobial
Source: PLoS Genet. 2025 Mar 7;21(3):e1011610. doi: 10.1371/journal.pgen.1011610 (PMC11918324; doi:10.1371/journal.pgen.1011610)
Supplement: Attachment — Submitted filename: PGENETICS-D-24-00732_Reviewer_response_20241205.pdf [file pgen.1011610.s026.pdf]

We thank the reviewers for their comments and have revised our manuscript extensively to address their comments. Specifically, we have performed additional experiments to test the effect of the *codY mutation in co-culture with *P. aeruginosa*, as well as against other *P. aeruginosa* produced toxins, and shown the data in new figures (Fig. 3, Supp. Fig. 4 and 15). Additionally, we have substantially edited our results section, especially the part describing the transcriptional responses, to make it more focused and easier to follow. We have also provided additional details about our evolution. We believe that these changes have considerably enhanced our manuscript. Our detailed point-by-point responses to the reviewers' comments are in bold below.**

Reviewer #1: Infections are often polymicrobial. Pseudomonas aeruginosa and Staphylococcus aureus are two common opportunistic pathogens of humans that are often co-isolated from infection sites. Given that P. aeruginosa typically outcompetes S. aureus in in-vitro experiments the question is how these two pathogens can co-exist in infections. In their paper, Martini and colleagues tackle this question and examine whether S. aureus can adapt to inhibitory compounds secreted by P. aeruginosa. They particularly focus on pyocyanin, a broad-spectrum toxin produced by P. aeruginosa. They used experimental evolution to repeatedly expose cultures of S. aureus to pyocyanin. They found that S. aureus evolved tolerance to this compound and that the evolved phenotypes were associated with mutations in codY, a global regulator. Subsequent analysis involving transcriptomics and work with defined mutants elucidate the role of CodY for pyocyanin protection. Given that CodY is a global regulator, many pathways are affected and the authors did a good job in going very deep to disentangle to various downstream effects that mutations in codY can have.

Overall, this is a strong study. The only main critic I have is that the manuscript is attractive to a general audience only at the beginning. Very soon, the analyses and narrative become specific and detailed, probably only of interest to a narrow group of specialists. I understand that many of the downstream analysis are required to understand the effects of mutations in CodY. Nonetheless, I (considering myself as an average reader) quickly lost interest after Fig. 2 and my willingness to navigate through all the transcriptomics comparisons and effects of individual mutants (shown in 3 main figures and 13 supplementary figures) quickly depleted. I cannot give direct advice of how to amend this problem, but I strongly encourage the authors to improve their narrative in the second half of their manuscript to better guide the reader through the complex data. Perhaps, "less is more" and a more focussed text (and narrative) would help.

As suggested by the reviewer, we have streamlined the narrative, substantially reducing the description of the transcriptomics, to help readers focus on the mechanistically relevant parts. We do have several supplementary figures that test (and disprove) various hypotheses about the putative PYO tolerance mechanism of the *codY mutant, but we thought it was important to retain those experiments (negative data) in the manuscript, to rule out those mechanisms. However, we have significantly condensed the narrative for these experiments.**

Further comments:

1. Experimental evolution 1. Some discussion on the specifics of the experimental evolution experiment would be helpful. For mutations to arise and spread, bacteria need to divide. It seems that PYO prevents bacterial growth. So, how can mutation and selection occur under such a

treatment? This question is important to address. There are at least two scenarios. First, there are a few cell divisions under PYO treatment and this allows mutants to arise and selection to operate. Second, mutation accumulation occurs during the recovery phase and selection happens under PYO treatment. The fact that 9 different mutations in codY were found (among 18 clones) strongly suggests that the second scenario occurred. Neutral mutations in codY occurred during the recovery phase and were then all selected during treatment. Conversely, under the first scenario one would expect the first emerging codY mutant to quickly spread. Thus, there should be a single codY mutant per population and not so many different ones.

We agree with the reviewer that mutations probably arose during growth and were then selected for in PYO. We have mentioned this in the results section (lines 139-141).

2. Experimental evolution 2. Additional information should be provided on the experimental design.

a. How many cell division occurred during the treatment and the recovery phase?

There was no increase in cell numbers during the treatment, and we have mentioned an estimate of the generations during the recovery phase (lines 112-114).

b. Why were there only two replicated populations? It is hard to derive general patterns with N=2.

c. There is no control treatment, in which S. aureus evolved without PYO. Such controls are typically used to differentiate between medium and treatment adaptations.

Our goal for the selections was to identify adaptations that led to increased survival specifically upon PYO exposure and not in media, and dissect the underlying mechanism(s). Our rationale to start with two independently PYO evolved populations, was to identify common mutations and then test them for a differential survival phenotype in both PYO and control conditions. The increased tolerance and survival of the CodY mutant specifically in PYO (and not in media) indicates that it was selected as a result of PYO exposure and not due to adaptation to the media. Further, the *codY mutant phenocopied the evolved isolates (in terms of PYO tolerance) suggesting that it was the major (if not only) contributor to PYO tolerance in our populations. Given that our 2 evolved populations allowed us to identify a specific PYO tolerance mutation and mechanism, we did not pursue additional PYO or control population evolutions.**

We agree with the reviewer that, in principle, additional populations, or control populations, can be useful to identify mutations critical for differential survival, but we did not deem it necessary for our study since we had already identified and validated the *codY* mutation from our 2 evolved populations. We have mentioned this now in the manuscript (lines 116-118, 154-156, and 552-556).

d. Why was there an imbalance between the number of colonies picked per population (11 vs. 7)?

We originally tested and sequenced seven colonies from both populations. Given that there was a wide diversity of mutations (including *codY* alleles) present in population A, we sequenced a further four colonies from population A to potentially identify additional mutations. We have mentioned this in the manuscript (lines 127-131).

3. This study exposes S. aureus to PYO and does not directly investigate evolutionary responses of S. aureus to P. aeruginosa. That is why it is important to provide some context on what might happen when the species truly interact.

We have now directly tested whether the *codY allele provides protection in co-culture with *P. aeruginosa* and found that the *codY** mutant shows increased tolerance compared to the WT in co-culture with *P. aeruginosa*, a phenotype that is likely dependent on pyocyanin production. This data is shown in Figure 3 and described on <u>lines 188-199</u>.**

a. How much PYO is typically produced by P. aeruginosa and how does natural production relate to the concentrations used in this study?

PYO concentrations in lab cultures and clinical samples (sputum, aspirates) range between 0 - ~150 µM, although higher values have also been observed, and given that local concentrations are likely to be higher, the concentrations we use are expected to be of biological and physiological relevance. We have now mentioned this in the manuscript (<u>lines 102-104</u>).

b. P. aeruginosa produces other phenazines in addition to PYO. Are similar evolutionary responses expected to these other phenazines?

c. Discussion on whether codY mutation are specific response to PYO or could also be beneficial to cope with other toxins produced by P. aeruginosa.

We directly tested whether the *codY mutation affects responses to other phenazines, as well as other *P. aeruginosa* toxins like rhamnolipids, siderophores, and alkylquinolones. Of the tested antimicrobials, we see substantial (~10-fold) increased survival of the *codY** mutant compared to the WT in HQNO, and minor (1.5-2-fold) survival differences for PQS and PCA, suggesting that this mutation provides relatively narrow protection against PYO and HQNO. This data is shown in Supp. Fig. 4 and described on <u>lines 178-187</u>.**

4. Please indicate early on what type of S. aureus strain was used.

We have now mentioned this early in the Results section (<u>line 100</u>).

5. Lines 198-199 and other places. Please remove priority claims. They are not helpful in any way.

We have carefully revised the text to remove all priority claims.

6. Lines 236-239 and other places. The transcriptomic comparisons are often very descriptive. In this specific section, the authors claim that "response ... was overall similar". What does that mean? I recommend to use statistical approaches to distinguish what was similar and what was different.

We used 'overall similar' because most of the same pathways were enriched in the upregulated and downregulated genes in both the WT and *codY* mutant. However, as mentioned above, we have extensively revised the description of this section to ensure that the text is much more focused. So that sentence has been removed.

Reviewer #2: This manuscript defines a new mechanism of resistance in Staphylococcus aureus (SA) to the antimicrobial pyocyanin (PYO). PYO is produced by Pseudomonas aeruginosa (PA) and is known to have the ability to kill SA. Here the Khare group use experimental evolution and detect

mutations in the gene encoding the SA transcriptional regulator CodY was responsible for tolerance to PYO. They go on to identify some of the factors regulated by CodY that may be important for this phenotype. However there are a number of major issues with the data and conclusions as presented:

The authors do not discuss whether the levels of PYO used for these have any biological relevance.

As mentioned above, PYO concentrations in lab cultures and clinical samples (sputum, aspirates) range between 0 - ~150 µM, although higher values have also been observed, and given that local concentrations are likely to be higher, the concentrations we use are expected to be of biological and physiological relevance. We have now mentioned this in the manuscript (lines 102-104).

For the graphs in Figure 1D and E, it is unclear if the different points at the different times represent replicates or different colonies from the same population.

In these figures the different points represent different isolates from the evolved populations. This has been clarified in the text (lines 119-122) and figure legend.

There seems to be no untreated WT population transferred similarly to the treated population.

As mentioned above, our goal for the selections was to identify adaptations that led to increased survival specifically upon PYO exposure and not in media, and dissect the underlying mechanism(s). Our rationale to start with two independently PYO evolved populations, was to identify common mutations and then test them for a differential survival phenotype in both PYO and control conditions. The increased tolerance and survival of the CodY mutant specifically in PYO (and not in media) indicates that it was selected as a result of PYO exposure and not due to adaptation to the media. Further, the *codY mutant phenocopied the evolved isolates (in terms of PYO tolerance) suggesting that it was the major (if not only) contributor to PYO tolerance in our populations. Given that our 2 evolved populations allowed us to identify a specific PYO tolerance mutation and mechanism, we did not pursue additional PYO or control population evolutions.**

We agree with the reviewer that, in principle, additional populations, or control populations, can be useful to identify mutations critical for differential survival, but we did not deem it necessary for our study since we had already identified and validated the *codY* mutation from our 2 evolved populations. We have mentioned this now in the manuscript (lines 116-118, 154-156, and 552-556).

Line 118-Apparently 18 terminal isolates (presumably this is after Treatment 7, but this is never stated) were sequenced, but tolerance of these isolates to PYO does not seem to be presented.

We presented tolerance data for a subset of these isolates in the main figures (Fig 1D and 1E) and have shown the rest in Supplementary Figure 1. We have also highlighted the populations from which the isolates were taken in Fig. 1C (treatment 5 for Population A and treatment 7 for Population B) and mentioned this in the figure legend and text (lines 550-552 and 1087-1088).

One of these CodYR222C (referred to as codY*) was selected for further study but whether all had the same level of tolerance or not does not seem to be shown.

We show the tolerance level for our evolved isolates that had different mutations in *codY*, and their tolerance phenotypes are likely due to the *codY* mutant alleles they carry. However, most of the isolates had additional mutations as well, and to cleanly test the effect of a *codY* mutation, we generated a clean allele replacement strain. We also tested a *codY*::tn mutant from a well-established transposon library. Given that the phenotype was due to loss of function of *codY*, we decided to further study the mutant we had created, and the mechanism of PYO tolerance, instead of generating additional allele-replacement strains.

It is unclear whether codY* is inactive (for all its known functions) or just less active.

While we cannot rule out that the *codY allele has some residual activity, the RNAseq data suggests that it is a hypoactive allele of CodY that mimics the dysregulation seen in a *codY* null mutant. We have mentioned this in the text (lines 212-215).**

A codY transposon mutant was used as a control, but why wasn't a complete codY deletion generated and tested?

The mutant is from the NTML library, which is a validated library widely used in the field. The standard in the field is to routinely use mutants from the library as null mutants. We have mentioned the library in the text (lines 151-154).

One mechanism of resistance to PYO is the generation of small colony variants (SCV) (line 402 and references 38 and 96), but whether any mutations in genes known to be mutated in SCV does not appear to be considered. Related to this, were SCV observed in the evolution study on the "enumerate CFU" plates (Figure 1B)?

We did not observe SCVs during the evolution and did not see any SCV related mutations in our study (Supplementary Data File 1). Given that there was a recovery phase in our selection, it is not surprising that we did not recover SCVs at the end of the selection process, as they are expected to have a significant growth defect in regular media. SCVs are thought to provide resistance to PYO, and it is possible that they do not contribute to PYO tolerance, which is the phenotype we are studying. We have added this consideration to the Discussion section (lines 431-435).

The authors noted some alterations in the coding sequence of CodY when they surveyed >60,000 SA genomes, but the frequency of these (and how it compares to other similar genes) does not seem to be presented. The information shown in Supp. Data File 04 does not seem to address this and what is being shown is very difficult to understand.

We showed the CodY mutations in *S. aureus* isolates to demonstrate that some of the mutations we identified do appear in natural isolates. We believe that any variation in the protein (compared to the lab JE2 strain) also includes natural variation that may not affect function, and thus comparing frequencies of changes to those seen in other genes will not be informative, without much more in-depth analysis. We have mentioned this in the manuscript (lines 369-371). We have also clarified the information present in Supp. Data File 04 in an 'Info tab' in the file.

The impact of the codY mutation of SA in response to PA does not appear to be presented.

As mentioned above, we have now directly tested whether the *codY allele provides protection in co-culture with *P. aeruginosa* and found that the *codY** mutant shows increased tolerance compared to the WT in co-culture with *P. aeruginosa*. This data is shown in Figure 3 and described on lines 188-199.**

Minor:

The discussion of a number of relevant papers seems to be missing including:

https://pubmed.ncbi.nlm.nih.gov/37227819/

https://pubmed.ncbi.nlm.nih.gov/38259061/

We have cited these papers appropriately in the manuscript (lines 404-405 and 59).
